# Enhancing access to alcohol use disorder pharmacotherapy and treatment in primary care settings: ADaPT-PC

**DOI:** 10.1186/s13012-016-0431-5

**Published:** 2016-05-10

**Authors:** Hildi J. Hagedorn, Randall Brown, Michael Dawes, Eric Dieperink, Donald Hugh Myrick, Elizabeth M. Oliva, Todd H. Wagner, Jennifer P. Wisdom, Alex H. S. Harris

**Affiliations:** 1 Veterans Affairs Health Services Research and Development Center for Chronic Disease Outcomes Research, Minneapolis Veterans Affairs Health Care System, Minneapolis, MN 55417 USA; 2 Department of Psychiatry, University of Minnesota School of Medicine, Minneapolis, MN 55455 USA; 3 William S. Middleton Memorial Veterans Hospital, Madison, WI 53705 USA; 4 Department of Family Medicine, University of Wisconsin School of Medicine and Public Health, Madison, WI 53715 USA; 5 Substance Abuse Treatment Program, South Texas Veterans Affairs Health Care System, San Antonio, TX 78229 USA; 6 Department of Psychiatry, University of Texas Health Science Center at San Antonio, San Antonio, TX 78229 USA; 7 Center for Drug and Alcohol Problems, Ralph H. Johnson Veterans Affairs Medical Center, Charleston, SC 29401 USA; 8 Department of Psychiatry and Behavioral Services, Medical University of South Carolina, Charleston, SC 29425 USA; 9 Veterans Affairs Health Services Research and Development Center for Innovation to Implementation, Palo Alto Veterans Affairs Health Care System, Menlo Park, CA 94025 USA; 10 Health Economics Resource Center, Palo Alto Veterans Affairs Health Care System, Menlo Park, CA 94025 USA; 11 Department of Health Policy, George Washington University, Washington, DC 20052 USA

**Keywords:** Alcohol use disorder treatment, Alcohol use disorder pharmacotherapy, Primary care mental health integration, Implementation

## Abstract

**Background:**

Only 7.8 % of individuals meeting diagnostic criteria for alcohol use disorder (AUD) receive treatment in a given year. Most individuals with AUDs are identified in primary care (PC) settings and referred to substance use disorders (SUD) clinics; however, only a minority of those referred attend treatment services. Safe and effective pharmacological treatments for AUD exist, but they are rarely prescribed by PC providers. The objective of this study is to refine, implement, and evaluate an intervention to integrate pharmacological AUD treatment options into PC settings. This paper provides a detailed description of the intervention design and the evaluation components.

**Methods/design:**

Three large Veterans Health Administration (VHA) facilities are participating in the intervention. The intervention targets stakeholder groups with tailored strategies based on implementation theory and prior research identifying barriers to implementation of AUD pharmacotherapy. Local SUD providers and primary care mental health integration (PCMHI) providers are trained to serve as local implementation/clinical champions and receive external facilitation. PC providers receive access to consultation from local and national clinical champions, educational materials, and a dashboard of patients with AUD on their caseloads for case identification. Veterans with AUD diagnoses receive educational information in the mail just prior to a scheduled PC visit. Effectiveness of the intervention will be evaluated through an interrupted time series with matched controls to monitor change in facility level AUD pharmacotherapy prescribing rates. Following Stetler’s four-phase formative evaluation (FE) strategy, FE methods include (1) developmental FE (pre-implementation interviews with champions, PC providers, and Veterans), (2) implementation-focused FE (tracking attendance at facilitation meetings, academic detailing efforts by local champions, and patient dashboard utilization), (3) progress-focused FE (tracking rates of AUD pharmacotherapy prescribing and rates of referral to PCMHI and SUD specialty care), and (4) interpretive FE (post-implementation interviews with champions and PC providers). Analysis of FE data will be guided by the Consolidated Framework for Implementation Research (CFIR).

**Discussion:**

If demonstrated to be successful, this implementation strategy will provide a replicable, feasible, and relative low-cost method for integrating AUD treatment services into PC settings, thereby increasing access to AUD treatment.

## Background

The ADAPT-PC protocol provides a detailed description of an intervention to improve access to evidence-based pharmacological treatments for alcohol use disorders (AUD) in primary care settings that was designed to be easily scalable and relatively inexpensive. The protocol also provides a detailed description of how implementation frameworks and methods can be used to inform the design, refinement, and evaluation of an implementation intervention.

In 2013 in the USA, 16.6 million adults (7.0 %) met diagnostic criteria for an AUD and 6.8 % of the population engaged in heavy drinking (five or more drinks on five or more days out of the past 30 days) [1]. AUDs and heavy drinking are associated with car crashes, domestic violence, neurocognitive impairments, poor medication adherence, psychiatric comorbidity, and increased morbidity and mortality [2–8]. The total societal costs of AUDs and heavy drinking, including health care costs, law enforcement costs, other direct costs (e.g., material losses due to accidents), and productivity losses were estimated in 2006 to be over $131 billion dollars, representing 1 % of the total US Gross Domestic Product [9]. Despite the high prevalence and costs associated with AUDs, treatment rates in the general population remain astonishingly low. In 2013, only 7.8 % of individuals meeting diagnostic criteria for AUD received any AUD treatment [6]. Improving access to evidence-based treatments for AUD has the potential to realize savings in health care costs given the multiple chronic conditions that are exacerbated by AUD and the high rate of accidental injury associated with heavy drinking and AUD.

The under-treatment of and costs associated with AUD are also major problems within the Veterans Health Administration (VHA). In fiscal year 2010, 360,729 VHA patients had an AUD diagnosis but only a third of these patients received treatment in one of VHA’s 220 specialty addiction programs [10]. Yu and colleagues found that the marginal yearly treatment cost (cost above the mean for the entire sample) associated with a diagnosis of an AUD was $3124 (1999 dollars) per patient [11].

Clearly, different models are needed to increase access to treatment for the majority of individuals with AUD that are not currently receiving treatment. Although the need is great and interest in the integration of AUD treatment into primary care settings is high, few models for implementation of AUD treatment into primary care have been tested. Pharmacotherapy for AUD using naltrexone or acamprosate is a vastly underutilized evidence-based treatment for AUD that may have more potential for integration into primary care settings relative to intensive behavioral or psychosocial treatments.

Randomized controlled trials and meta-analyses support the efficacy of pharmacological treatment with naltrexone or acamprosate to improve drinking outcomes including time to relapse, drinking days, and number of drinks per drinking day [12–15]. Use of naltrexone or acamprosate for patients with AUD is supported by National Quality Forum’s National Voluntary Consensus Standards for the Treatment of Substance Use Conditions published in 2007 and the VA-Department of Defense (DoD) Clinical Practice Guidelines (CPG) for Management of Substance Use Disorders (SUD) updated in 2009.

Despite the evidence supporting the use of naltrexone and acamprosate for treating AUD, these medications are underutilized in the USA [16]. While over 11 million individuals in the USA met criteria for AUD in 2006 [6], only an estimated 674,000 prescriptions were filled for AUD medications (includes disulfiram as well as naltrexone and acamprosate) [16]. If each prescription was for a unique individual (a highly unlikely scenario), then only 6 % received pharmacotherapy.

Among the 440,000 VHA patients with a documented AUD diagnosis in fiscal year 2012, only 5.8 % received any approved AUD pharmacotherapy. There is also extreme variability in prescribing rates at the facility level. The rate of pharmacotherapy for AUD among Veterans who received AUD specialty care ranged from 0 to 20 % by facility with substantially lower prescribing rates for Veterans with no contact with AUD specialty care [17]. Extremely low prescribing rates and significant variation across facilities suggests that significant gaps exist in access to these medications and that more could be done to increase prescribing rates.

### Diagnosing the causes of quality/performance gaps

Previous work conducted by team investigators has indicated that the most predominant barriers to implementation of AUD pharmacotherapy include (1) perceived low patient demand, (2) pharmacy or formulary restrictions, (3) inadequate provider training in the use of AUD medications, (4) lack of provider confidence in the effectiveness of AUD medications, and (5) lack of patient awareness of AUD medications [18, 19]. This foundational work has led to the identification of key strategies to overcome these barriers and promote implementation of AUD pharmacotherapy including (1) educating providers about the effectiveness and appropriate use of AUD medications, (2) educating patients about AUD medication options, (3) activating patients to discuss AUD medication options with their providers, (4) increasing physician involvement in AUD treatment, (4) identifying, training, and supporting local role models or champions, and (5) facilitating connections between providers and local AUD specialists. This paper describes the development of an implementation trial to increase AUD pharmacotherapy prescribing rates in primary care.

## Methods/design

This trial will examine the feasibility, acceptability, and effectiveness of an implementation intervention to improve rates of AUD pharmacotherapy prescribing in primary care settings in three VHA facilities. The intervention includes training local clinical champions to provide education and support to primary care providers, an educational website that includes access to provider-specific dashboards for case identification, and mailing of educational materials to identified veterans. The primary outcome is prescribing rates which will be examined through an interrupted time-series analysis with matched control comparisons. Formative evaluation and a cost assessment will assess feasibility and acceptability of the intervention and inform quantitative results.

### Theoretical model

The theory of planned behavior was selected to further guide the development of our intervention components because the trial was designed to change individual behaviors: physicians’ decisions to discuss and prescribe AUD medications and patients’ decisions to discuss AUD pharmacotherapy with their provider [20]. The theory of planned behavior hypothesizes that intention to act is driven by one’s *attitude toward the behavior*, one’s *subjective perception of peer norms* related to the behavior, and one’s *perceived behavioral control* (ability to perform the behavior). Combining the recommendations from the foundational description of barriers with the theory of planned behavior, we selected the strategies described in Table 1 for inclusion in our multifaceted implementation intervention.

**Table 1:** Multifaceted implementation intervention strategies

Local clinical champions	Primary care providers	Veterans
• 1.5-day collaborative learning session • Monthly facilitation teleconferences • On-demand access to national clinical and implementation champions • Access to website with patient and provider educational materials • Access to patient dashboard at facility level	• Educational outreach provided by local champions in large group (Grand Rounds) and small group (practice team) settings • On-demand access to national and local clinical champions • Access to website with patient and provider educational materials • Access to patient dashboard at the provider level • Reminder emails when new patients added to provider level dashboard	• Patient educational materials mailed to home address

### Selection and recruitment of sites

Three large VHA medical facilities were recruited to participate in this trial. Sites were chosen based on the availability of SUD specialty care providers and Primary Care Mental Health Integration (PCMHI) providers interested in training as local clinical champions. While participating sites were somewhat above average in overall prescribing rates of AUD pharmacotherapy for VHA medical facilities, very little of that prescribing was occurring outside of SUD specialty clinic settings where only a fraction of patients with AUD diagnoses receive treatment, indicating plenty of room for improvement in our targeted population (Veterans with AUD seen in primary care clinics).

### Phase 1: developmental formative evaluation

To understand patient perspectives on pharmacological treatments for AUD and barriers and facilitators to their use of pharmacological treatments, ten veterans with self-identified alcohol misuse will be recruited for interviews at each site. Interview questions will be guided by the theory of planned behavior to address attitudes toward AUD medications (including their perceived safety and efficacy), perceived peer norms regarding AUD medications, and perceived ability to discuss AUD medications with their provider and use the medications consistently and appropriately. Veterans will also have the opportunity to review the patient educational materials and will be queried regarding their likely response to receiving educational materials in the mail, any privacy concerns they may have with this plan, alternative methods of making the materials available, and any suggestions for additional information/materials that they would find helpful.

To understand primary care providers’ perspectives on pharmacotherapy for AUD and barriers and facilitators to prescribing pharmacotherapy for AUD in primary care settings, eight to ten primary care providers will be recruited for interviews at each site. Interview questions will be guided by the theory of planned behavior to address knowledge of and attitudes toward the use of AUD medications, perceived peer norms regarding prescribing AUD medications, and perceived ability to integrate AUD pharmacotherapy into their practice. Interview questions will also address Consolidated Framework for Implementation Research (CFIR) constructs to identify local barriers and facilitators to implementation that will need to be addressed by the intervention [21]. Providers will also have an opportunity to review the provider educational materials to provide their input as to which tools and what formats of dissemination would be most helpful. Finally, providers will have an opportunity to review the patient educational materials and will be informed of the intent to mail the materials to patients so that they can express any concerns related to this strategy or suggest modifications to the strategy.

To understand how to best assist clinical champions in their role, each site’s participating local clinical champions (SUD specialty care prescriber and PCMHI provider) will complete brief interviews to provide input into the final planning of a collaborative learning session which will serve as the starting point of the intervention. Questions will assess whether and how prescribers currently use AUD medications in their practice, what they want to learn about AUD pharmacotherapy, what formats they prefer for learning, and what they feel they would need to learn to provide support to primary care prescribers. These interviews will also include questions related to CFIR constructs for the purpose of identifying local barriers and facilitators to implementation.

Qualitative data collected during developmental interviews will be analyzed using rapid qualitative analysis techniques so that the data can be used to inform the final details of the intervention plan [22].

### Phase 2: three site feasibility trial of a multifaceted implementation intervention

#### Site champion intervention

The site champion intervention incorporates empirically based education and implementation methods including participatory and interactive education, follow-up, and performance feedback [23–28].

Two site champions from each site (a SUD specialty care prescriber and PCMHI provider) will be contacted by the Project Coordinator to complete informed consent and their pre-training interview (describe above). Once interviews are complete, we will schedule the in-person collaborative learning session. All efforts will be made to ensure that both participants from each facility are available to attend. While the session will include a didactic component, the majority of the session will be collaborative and involve group discussions. The goals of the in-person collaborative session are to ensure that all champions are knowledgeable about (1) identifying risky/heavy drinking and AUDs, (2) brief interventions for risky/heavy drinking, and (3) pharmacological and behavioral treatment options for AUDs. Additional goals are to (1) increase champions’ capability to facilitate the consideration of AUD pharmacotherapy by primary care prescribers at their site and (2) assist champions in identifying local options for providing disease care management for AUD patients receiving pharmacotherapy.

Following the in-person collaborative learning session, attendees will receive external facilitation in the form of monthly teleconference meetings for 9 months to review the agreed upon outreach plans, monitor progress, and identify implementation barriers and strategies to overcome them. Additional resources such as an email group and access to an external expert consultant upon request will also be available during that time. Finally, the site champions will receive quarterly feedback reports on the prescribing rate at their site as well as at the other participating facilities. The external facilitators will address the feedback reports and help champions interpret and apply the information learned.

#### Primary care prescriber intervention

Veterans at each site who have received a diagnosis of AUD or an Alcohol Use Disorders Identification Test—Consumption Questions (AUDIT-C) score of greater than 8 in Outpatient Patient Care Encounter files within the past 12 months, do not have an active prescription for an AUD medication (naltrexone, acamprosate, or disulfiram), and have an upcoming primary care appointment within the next 30 days will be identified on a rolling basis (once per month for 9 months) for inclusion in the cohort. Once a veteran is included, their primary care provider will be identified and the provider’s electronic contact information will be collected for the purposes of providing real-time case identification for primary care providers.

On a weekly basis during the 9-month intervention period, the project web application will automatically send email invitations to primary care prescribers at the intervention sites that have a patient in the target cohort scheduled in their clinic within the next 7 days. The email will describe the project goals and design. It will provide a link to the project website where prescribers will have access to patient and provider educational materials and contact information for the national clinical champions and the local SUD specialty care and PCMHI champions. The provider will also be able to click on a link to access the list of identified patients on their panel with appointments upcoming in the next 7 days. Following the initial email, primary care providers will receive an email alert when they have a new upcoming appointment with a patient with an AUD diagnosis.

#### Veteran intervention

Each month, veterans newly added to the cohort will be mailed educational materials. Veterans will only be mailed materials once during the intervention even if they qualify for the cohort during more than 1 month. Patients will be sent a cover letter presenting the mailing as general educational information for VHA patients including “tips” for planning a conversation about their alcohol use with their primary care provider and an educational brochure titled Medication-Assisted Treatment for Alcohol Dependence developed by the VHA Office of Informatics and Analytics as part of a toolkit to support implementation of the VA/DoD Substance Use Disorders Clinical Practice Guideline (http://www.healthquality.va.gov/guidelines/MH/sud/SUDTool3PatientBookletFinalHiRes.pdf).

### Evaluation

The evaluation includes (1) the quantitative evaluation of the impact of the intervention on the primary outcome of AUD pharmacotherapy prescribing rate, (2) a formative evaluation designed using Stetler’s four-phase formative evaluation model [29], and (3) a cost assessment.

#### Effectiveness of the implementation intervention

The primary evaluation of the intervention’s effectiveness will use an interrupted time-series design to compare post-intervention outcomes and trends to pre-intervention levels within the three intervention sites. This approach is most powerful and provides the best control for facility characteristics, because sites are compared to themselves. However, this approach is vulnerable to confusing intervention effects with secular trends or disruptions that affect all sites in the system. Therefore, we will conduct sensitivity analyses using comparison sites as controls. For each intervention site, candidate comparison sites will be identified by closely matching on several variables including SUD specialty care and non-specialty care AUD prescribing rates, overall AUD diagnosis rates, and facility size. One site will be randomly selected from the identified close matches for each intervention site. Monitoring of prescribing trends will occur exclusively through the use of available administrative data. No intervention or other activities will occur at the comparison sites.

#### Primary hypothesis 1

The rate of receipt of AUD medications among directly targeted patients will increase in magnitude after the onset of the intervention.

The primary outcome will be the proportion of veterans diagnosed with AUD on an encounter within the past 12 months who have an upcoming primary care appointment who fill a prescription for AUD medications within 1 month of the targeted appointment. This rate will be calculated monthly for a 16-month pre-intervention period and then during the 9-month intervention period. Because the optimal timeframe for observing the desired outcome is unknown, sensitivity analyses will be conducted on the follow-up duration (e.g., 2- and 3-month post-primary care visit). Using a mixed-effects, segmented logistic regression analysis, we will evaluate if there are changes in either the level or slope following the onset of the intervention.

Then, we will elaborate this model to add the comparison to the control sites. This helps protect against the threat of misinterpreting secular effects occurring in the overall system as intervention effects. We will also conduct several other models to fully understand the effects of this intervention, including models that (1) incorporate separate “start-up” (i.e., months 17–20) and evaluation (i.e., months 18–25) segments and (2) look beyond month 25 to characterize a segment for post-intervention sustainment.

#### Primary hypothesis 2

The rates of consultations made from primary care to AUD specialty care and PCMHI among directly targeted patients will increase in magnitude after the onset of the intervention.

This analysis will be completely parallel to hypothesis 1 except the outcome will be the occurrence of a consultation to AUD specialty care or PCMHI for the targeted patients following the index primary care visit. Data on consultations will be extracted from the consultation table in the VA Corporate Data Warehouse, from which we can identify the requesting and requested specialties and providers.

#### Secondary hypothesis 1

The rate of receipt of AUD medications will increase in magnitude after the onset of the intervention for patients with AUD who are *NOT* targeted by the intervention.

The intervention may work by different pathways and influence the treatment received by patients who are not directly targeted by the intervention (sent educational materials prior to a primary care visit), especially through the prescribing and other efforts of the trained local champions. Therefore, we will evaluate trends and changes in AUD medication prescribing rates in patients diagnosed with AUD on an encounter within the past 12 months who have an upcoming *non-primary care mental health or AUD clinic appointment* (and who are *not* sent pre-primary care visit educational materials) who then fill a prescription for AUD medications within 1 month of the targeted appointment. These analyses will exclude the targeted patients. The sampling and analysis for this hypothesis will be completely parallel to hypothesis 1, except it is focused on the non-targeted population, i.e., patients with AUD who have appointments in AUD or mental health clinics, but not primary care.

#### Secondary hypothesis 2

The rate of active consideration of AUD medications, as measured by chart review, will increase in magnitude after the onset of the intervention.

Although the main goal of the intervention is to increase AUD pharmacotherapy prescribing rates, it is assumed that there will be a portion of patients who discuss pharmacological treatment options with their provider but do not start a prescription for some reason. It is difficult to confirm the occurrence of such a conversation with administrative data; data on consideration may be buried in progress notes, if it is documented at all. Given that there is currently little information available in the literature regarding the proportion of patients that *do not* receive an AUD medication after a process of active consideration, we will conduct a parallel outcome analysis (same approach as the primary hypothesis) except the outcome will be chart review determination of whether active consideration of these medications occurred even if a prescription was never filled in a subsample of patients. This will provide some data on whether the intervention had an impact on rates of consideration and, if so, how the increase in rates of consideration compared to the increase in rates of receipt. For a random sample of 50 included patients each month, we will use chart reviews to assess for rates of AUD pharmacotherapy consideration as documented in the progress notes of the targeted primary care visit. This will also provide some preliminary data to address the question of what proportion of patients would be expected to receive a prescription if routine consideration was in place and what proportion of patients would reject AUD pharmacotherapy after discussing the option with their provider. The chart review data will also provide information on reasons why a discussed pharmacological treatment option is not prescribed.

#### Formative evaluation

Formative evaluation involves a “rigorous assessment process designed to identify potential and actual influences on the progress and effectiveness of implementation efforts” [29]. Formative evaluation can serve many purposes over the course of an implementation project including identifying modifications to implementation efforts that may optimize opportunities for success, fostering an understanding of the causal events leading to change and the specific components of the intervention that most influenced outcomes, and informing future similar implementation efforts [29]. Our formative evaluation plan is guided by the four-phase model of formative evaluation identified by Stetler [29] which include the developmental, implementation-focused, progress-focused, and interpretive phases described below. Table 2 provides a summary of data collection methods and links each data source to the stage of formative evaluation that it primarily informs.

**Table 2:** Data collection methods for each formative evaluation stage

	Developmental	Implementation-focused	Progress-focused	Interpretive
Pre-intervention patient interviews	X			
Pre-intervention provider interviews	X			X
Primary care email read receipts and website access		X		X
Monthly facilitation meeting attendance		X		X
Monthly facilitation meetingotes			X	X
Quarterly feedback reports			X	X
Post-intervention provider interviews		X		X

#### Developmental formative evaluation

Developmental formative evaluation occurs prior to the start of an implementation intervention to determine the feasibility of the proposed implementation intervention and to solicit input from stakeholders regarding strategies for improving the intervention plan [29]. For the current study, as described above, developmental formative evaluation information will be collected through pre-intervention veteran, primary care prescriber, SUD specialty care, and PCMHI provider interviews.

#### Implementation-focused formative evaluation

Implementation-focused formative evaluation occurs during the process of implementation and focuses on the discrepancies between the implementation plan and its execution in order to document actual implementation processes and evaluate and measure actual exposure to the intervention [29]. For the current project, examples of processes that will be documented include (1) champion attendance at the monthly facilitation meetings, (2) the percentage of identified primary care prescribers that opened study emails and accessed links to the study website and personalized feedback reports, and (3) the number and types of outreach activities with primary care prescribers that were initiated by the champions.

#### Progress-focused formative evaluation

The purpose of progress-focused formative evaluation is to monitor progress in terms of achieving implementation goals and performance targets to identify blocked progress, allowing steps to be taken to optimize the momentum of the intervention [29]. For the current study, progress-focused formative evaluation data will primarily come from the follow-up facilitation meetings. Meetings will focus on barriers to implementation and strategies to address identified barriers. Non-attendance of participants from a particular site will be documented, and outreach through individual calls or emails will be initiated to assess for stalled progress and offer assistance with barriers. Additionally, quarterly feedback reports on prescribing rates developed for participants will be reviewed by project staff to assess for any outliers, either low or high progress, so that individual outreach or positive feedback can be initiated.

#### Interpretive formative evaluation

Interpretive formative evaluation uses the information collected from all of the other formative evaluation stages as well as information collected at the end of the project regarding the experiences of participants to clarify the meaning of successful or failed implementation and to enhance understanding of an implementation strategy’s impact [29]. Additional information that will be collected at the end of the implementation intervention are follow-up interviews with the participating champions and a sample of primary care providers. The follow-up interviews will be guided by the CFIR which provides a systematic framework for identifying potential barriers and facilitators to implementation [21].

#### Formative evaluation data analysis techniques

While some formative evaluation data will be analyzed using simple counts, e.g., number of facilitation meetings attended and number of PC providers accessing their dashboard, formative evaluation data will primarily be analyzed using qualitative data analysis techniques. Qualitative data will include the veteran interview transcripts, facilitation meeting notes, and the pre- and post-intervention provider interview transcripts. All documents will be uploaded into a qualitative data analysis program (NVivo) that enables researchers to mark blocks of text with thematic codes and explore relationships among and between codes and participant groups. Qualitative data will be coded thematically following techniques described by Miles and Humberman [30]. Three research team members will independently review the transcripts and develop an initial coding list. A deductive analysis approach will be used during the development of the initial coding list to focus codes on pre-determined domains of interest including barriers to implementation, facilitators to implementation, strategies, and recommendations for the improvement of the intervention, and relevance to CFIR constructs. Following development and refinement of the initial coding list, coders will code each transcript using the list, and they will also add open codes (inductive coding) to identify important themes not represented by the pre-determined domains. Consensus meetings will be held for review of consistency in coding. Inconsistencies will be resolved through mutual discussion and agreement. Finally, representative quotes and associated codes that represent key themes will be identified through consensus with the research team.

#### Cost assessment

Studies have shown that AUD is associated with high costs and that pharmacotherapy treatment can be cost-effective compared to referral to AUD specialty care [31]. Despite strong and uniform research support for the effectiveness of pharmacotherapy to treat AUD, and the cost effectiveness of the treatment itself, a low-cost, effective, and easily adopted intervention to improve utilization has yet to be identified. The proposed intervention was designed to be easily scalable and relatively inexpensive to implement.

First, we will estimate the costs of the intervention, which will include the cost of the provider collaborative learning session, the staff costs to host conference calls and provide expert consultation, and the costs of distributing educational materials. We will track the time spent providing the patient and clinician components. We will also track the cost of supplies and travel. We will convert the time parameters into costs using national VHA wages [32].

Second, we will compare the cost of the intervention to the patients’ health care costs in a budget impact analysis. The goal of this analysis is to investigate how the intervention would affect a facility’s 1- and 2-year budgets. We expect that the intervention will have a very low marginal cost per patient but believe that it is important to document the cost for future implementation work.

For the budget impact analysis, we will follow VHA and national recommendations [33, 34] and use VHA administrative data. We will segment the utilization and cost data into categories of care [35] so that we can understand the budgetary impact on primary care, mental health specialty care, SUD specialty care, and pharmacy costs. The budget impact analysis will utilize an interrupted time-series design to understand how the intervention affects downstream utilization. This econometric approach to estimating net benefits was discussed by Hoch [36], and we used it to examine the return on investment for SUD spending [37].

### Trial status

Pre-intervention interviews have been completed. The champion collaborative learning session has taken place, and champions have engaged in local outreach activities with their primary care providers. The 9-month intervention phase, which includes monthly veteran mailings and weekly primary care provider email alerts along with the availability of on-demand consultation from local and national clinical champions, is currently underway.

## Discussion

The ADAPT-PC protocol provides a detailed description of an implementation intervention to improve access to evidence-based pharmacological AUD treatments in primary care settings as well as providing a detailed description of how implementation frameworks and methods can be used to enhance the design, refinement, and evaluation of an implementation intervention. If this implementation intervention is successful, it will provide a relatively low-cost, scalable model that builds on local expertise and can be disseminated to additional VHA medical facilities and elsewhere. The information gained from this project is highly likely to be of interest to health care systems beyond the VHA system which are also seeking methods to integrate AUD treatment into primary care settings. Increasing patient access to evidence-based AUD treatment has the potential not only to improve patient outcomes but also to realize savings in health care costs given the multiple chronic conditions that are exacerbated by AUD and the high rate of accidental injury associated with heavy drinking and AUD. If successful, the intervention may also serve as a model for interventions designed to increase implementation of other evidence-based psychiatric interventions.

This project will also contribute to the growing body of research examining the relationship between CFIR constructs and implementation success. With the formative evaluation data we are collecting, we will be able to examine whether key barriers and facilitators were consistent across sites or unique to sites, whether certain facilitators were necessary for successful implementation or, conversely, whether certain barriers were predictive of implementation failure. We will also be able to examine which pre-implementation barriers were mitigated by the intervention and which were not addressed. Such information will inform targeted enhancements to the intervention and possibly allow for tailoring of the implementation strategy to pre-identified barriers in future sites.

### Limitations

The main limitation to this study is that it includes only three medical centers, all within the VHA health care system. In addition, these sites were selected partially because there was existing expertise in AUD pharmacotherapy within the local substance use disorder treatment clinics. Each site also had local PCMHI providers that were interested in assisting with implementation. Therefore, generalizability to VHA medical centers without local AUD pharmacotherapy expertise, without PCMHI in place and willing to assist, or to medical centers outside of the VHA system may be limited.
